# Alternative and complementary therapies in osteoarthritis and cartilage repair

**DOI:** 10.1007/s40520-020-01515-1

**Published:** 2020-03-13

**Authors:** N. R. Fuggle, C. Cooper, R. O. C. Oreffo, A. J. Price, J. F. Kaux, E. Maheu, M. Cutolo, G. Honvo, P. G. Conaghan, F. Berenbaum, J. Branco, M. L. Brandi, B. Cortet, N. Veronese, A. A. Kurth, R. Matijevic, R. Roth, J. P. Pelletier, J. Martel-Pelletier, M. Vlaskovska, T. Thomas, W. F. Lems, N. Al-Daghri, O. Bruyère, R. Rizzoli, J. A. Kanis, J. Y. Reginster

**Affiliations:** 1 MRC Lifecourse Epidemiology Unit, University of Southampton, Tremona Road, Southampton, SO16 6YD UK; 2 NIHR Musculoskeletal Biomedical Research Unit, University of Oxford, Oxford, UK; 3 Bone and Joint Research Group, Centre for Human Development, Stem Cells and Regeneration, Institute of Developmental Sciences, University of Southampton, Southampton, UK; 4 Nuffield Department of Orthopaedics, Rheumatology and Musculoskeletal Sciences, University of Oxford, Oxford, UK; 5 Department of Physical and Rehabilitation Medicine & Sports Traumatology, FIFA Medical Centre of Excellence, IOC Research Centre for Prevention of Injury and Protection of Athlete Health, FIMS Collaborative Center of Sports Medicine, University Hospital and University of Liège, Liege, Belgium; 6 Rheumatology Department, AP-HP, Saint-Antoine Hospital, 4 Blvd. Beaumarchais, Paris, France; 7 Research Laboratory and Academic Division of Clinical Rheumatology, Department of Internal Medicine, University of Genoa, Genoa, Italy; 8 WHO Collaborating Centre for Public Health Aspects of Musculoskeletal Health and Aging, Liege, Belgium; 9 Leeds Institute of Rheumatic and Musculoskeletal Medicine, University of Leeds & NIHR Leeds Biomedical Research Centre, Leeds, UK; 10 Department of Rheumatology, Sorbonne Université, INSERM CRSA, AP-HP Saint-Antoine Hospital, Paris, France; 11 Centro Hospitalar de Lisboa Ocidental- Hospital Egas Moniz, Lisbon, Portugal; 12 CEDOC / NOVA Medical School, Nova University of Lisbon, Lisbon, Portugal; 13 Department of Surgery and Translational Medicine, University of Florence, Florence, Italy; 14 Department of Rheumatology and EA 4490, Lille University Hospital, Lille, France; 15 National Research Council, Neuroscience Institute, Aging Branch, Padua, Italy; 16 Department of Orthopaedic Surgery, Themistocles Gluck Hospital, Ratingen, Germany; 17 Faculty of Medicine, Clinic for Orthopedic Surgery and Traumatology, Clinical Center of Vojvodina, University of Novi Sad, Novi Sad, Serbia; 18 Institute of Outdoor Sports and Environmental Science, German Sport University, Cologne, Germany; 19 Osteoarthritis Research Unit, University of Montreal Hospital Research Centre (CRCHUM), Montreal, QC Canada; 20 Medical Faculty, Department of Pharmacology, Medical University Sofia, 2, Zdrave Str, 1431 Sofia, Bulgaria; 21 Department of Rheumatology, Hôpital Nord, CHU de Saint-Etienne, Saint-Étienne, France; 22 INSERM U1059, Université de Lyon, Saint-Étienne, France; 23 Location VU Medical Center, Department of Rheumatology and Immunology, Amsterdam University Medical Center, Amsterdam, The Netherlands; 24 Chair for Biomarkers Research, Biochemistry Department, College of Science, King Saud University, Riyadh, Kingdom of Saudi Arabia; 25 Division of Bone Diseases, Geneva University Hospitals and Faculty of Medicine, Geneva, Switzerland; 26 Mary McKillop Health Institute, Australian Catholic University, Melbourne, Australia; 27 Centre for Metabolic Bone Diseases, University of Sheffield Medical School, Sheffield, UK; 28 Department of Public Health, Epidemiology and Health Economics, University of Liège, CHU Sart Tilman B23, 4000 Liege, Belgium

**Keywords:** Osteoarthritis, Cartilage, Alternative, Therapy, Treatment, Herbal

## Abstract

Osteoarthritis (OA) is the most common joint condition and, with a burgeoning ageing population, is due to increase in prevalence. Beyond conventional medical and surgical interventions, there are an increasing number of ‘alternative’ therapies. These alternative therapies may have a limited evidence base and, for this reason, are often only afforded brief reference (or completely excluded) from current OA guidelines. Thus, the aim of this review was to synthesize the current evidence regarding autologous chondrocyte implantation (ACI), mesenchymal stem cell (MSC) therapy, platelet-rich plasma (PRP), vitamin D and other alternative therapies. The majority of studies were in knee OA or chondral defects. Matrix-assisted ACI has demonstrated exceedingly limited, symptomatic improvements in the treatment of cartilage defects of the knee and is not supported for the treatment of knee OA. There is some evidence to suggest symptomatic improvement with MSC injection in knee OA, with the suggestion of minimal structural improvement demonstrated on MRI and there are positive signals that PRP may also lead to symptomatic improvement, though variation in preparation makes inter-study comparison difficult. There is variability in findings with vitamin D supplementation in OA, and the only recommendation which can be made, at this time, is for replacement when vitamin D is deplete. Other alternative therapies reviewed have some evidence (though from small, poor-quality studies) to support improvement in symptoms and again there is often a wide variation in dosage and regimens. For all these therapeutic modalities, although controlled studies have been undertaken to evaluate effectiveness in OA, these have often been of small size, limited statistical power, uncertain blindness and using various methodologies. These deficiencies must leave the question as to whether they have been validated as effective therapies in OA (or chondral defects). The conclusions of this review are that all alternative interventions definitely require clinical trials with robust methodology, to assess their efficacy and safety in the treatment of OA beyond contextual and placebo effects.

**Electronic supplementary material:**

The online version of this article (10.1007/s40520-020-01515-1) contains supplementary material, which is available to authorized users.

## Introduction

Osteoarthritis (OA) is the most common form of arthritis and the global prevalence of knee OA alone is 3.8%, affecting over 250 million individuals worldwide [1]. OA is an increasingly major socioeconomic and public health issue, with the years lived with disability increasing by 64% from 1990 to 2010.

The current dogma is that OA may have differing causes but with a common, multi-tissue morphology including cartilage fibrillation, fissure and loss, subchondral bone changes and synovitis. OA is more prevalent in females than males and, although it can affect any joint, the most common anatomical sites include the knee, distal interphalangeal joints and hip [2]. Clinically, OA is characterized by joint pain, significant stiffness and leads to functional decline and a reduced quality of life for the affected individual.

There are a number of different treatments for OA including non-pharmacological and pharmacological approaches. However, despite a number of well-written and well-considered guidelines [3−6], there is no direct advice regarding the application of what may be termed ‘alternative’ treatments including autologous chondrocyte implantation (ACI), autologous/heterologous mesenchymal stem cells (MSCs), platelet-rich plasma (PRP), vitamin D and other therapies (e.g. oral collagens, methylsulfonylmethane, curcumin, ginger). This lack of appropriate clinical advice and information is an issue for clinicians when considering how best to advise patients, especially as some of these therapies have a high profile in the lay press.

A current literature review was, therefore, performed and a working group of the European Society for Clinical and Economic Aspects of Osteoporosis and Osteoarthritis (ESCEO) was convened to review, evaluate and summarize current evidence regarding these putative OA treatments, and to provide expert opinion on their current role in the treatment of OA. We have classified the alternative therapies into surgical and medical approaches.

## Surgical therapy for cartilage loss

Joint replacement is an established surgical technique focused on treating the end-stage of OA. For this reason, more minor surgical procedures have been developed to be used in the case of localized, traumatic or early disease with the aim of regenerating cartilage and rejuvenating the joint. In this section, we examine the evidence for the use of autologous stem cell and cartilage therapies as potential treatment options.

### Autologous chondrocyte implantation in knee cartilage defects

**Fig. 1:**
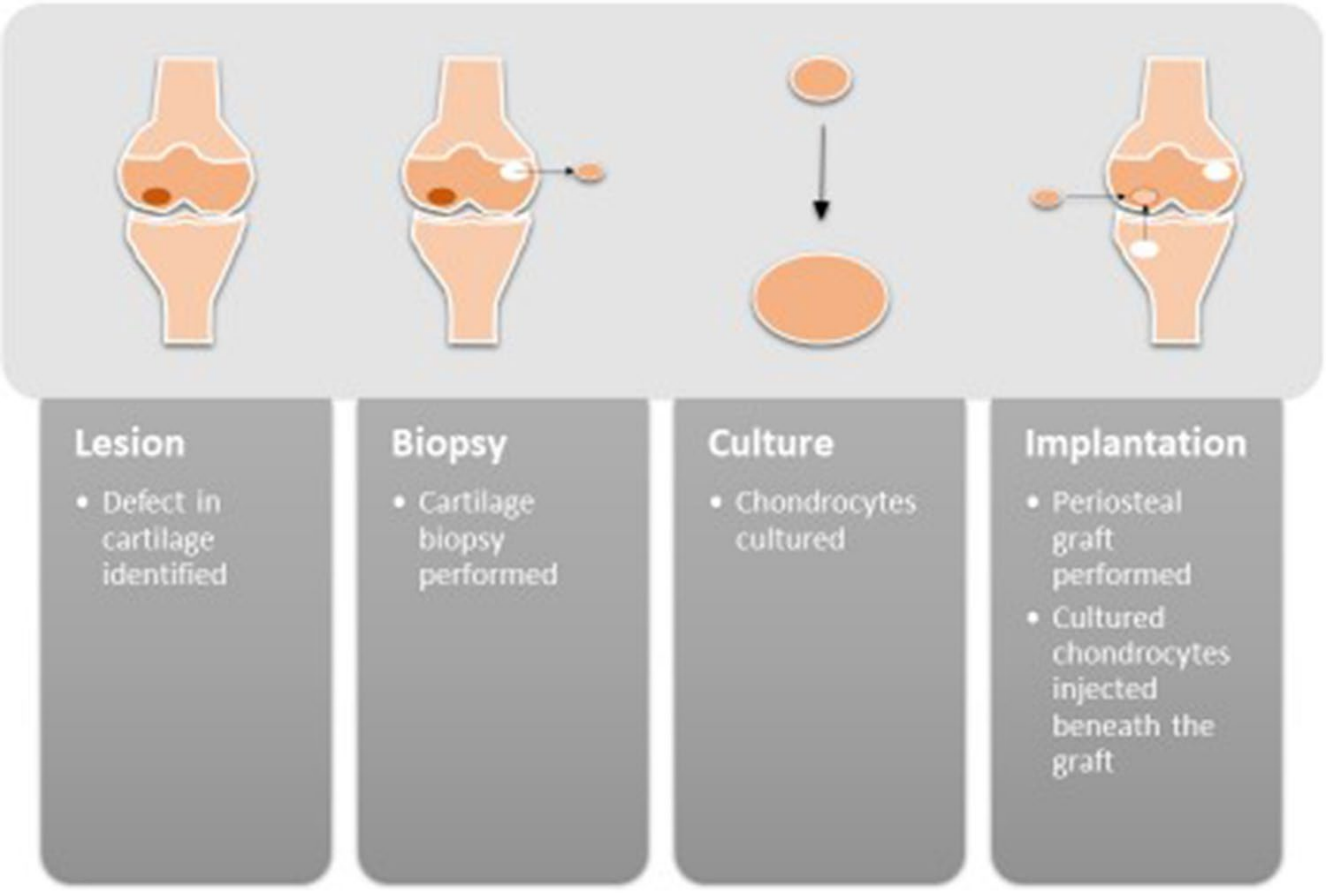
A schematic demonstrating the process of autologous chondrocyte implantation. A chondral lesion is identified and a biopsy of non-articular cartilage is performed. The biopsy is cultured to amplify the number of chondrocytes. These are then injected under a periosteal flap (which is acquired from the proximal tibia)

ACI has a 30 year history [7] and is an established technique for the treatment of ulcerated cartilage and cartilage defects. It involves an initial cartilage biopsy, from which chondrocytes are cultured in vitro. In a second surgical procedure, a flap or membrane is then sutured (or glued) over the defect and the cultured chondrocytes are injected under this barrier. This process is summarized in Fig. 1. Over the last 10–15 years ACI has evolved (as bioengineering technology has improved) and now includes matrix-assisted ACI (MACI). The patient then undergoes very careful and graded rehabilitation to prevent the patch being dislodged.

Early randomised controlled trials (RCTs) data suggested no significant benefit of ACI when compared to the alternative surgical option of microfracture [8] and although a histologic improvement was observed [9], the clinical relevance of this is questionable. Indeed a Cochrane review in 2011 concluded that there was insufficient evidence to recommend the use of ACI [10].

The method developed over time to include collagen-covered ACI, and subsequently MACI, with the latter providing benefits including reduced size of the incision, greater surgical consistency, more consistent cell seeding, reduced periosteal hypertrophy and fewer adverse events [11−14].

Indeed, the matrix-applied method did perform significantly better than microfracture in the SUMMIT study (an RCT of 144 patients over 2 years) [15] in terms of clinical and functional outcomes. There was however no significant benefit over microfracture in MRI or histological outcomes. A key finding from these RCTs is that there was no correlation between the functional outcomes and evidence of structural repair when MRI is used, which is a potentially concerning finding.

ACI in combination with meniscal transplant allograft has good long-term outcomes with 75% still functioning well at 10-years (and 25% proceeding to arthroplasty). It is difficult to delineate whether the benefits of the procedure are due to ACI, meniscal transplant or indeed osteotomy (performed as part of the procedure) [16]. Similar results have been demonstrated in 57 patient with bipolar chondral lesions in the tibiofemoral compartment [17] with 75% having no radiographic progression at 10 years.

The cost of ACI and MACI are high, ranging from £4125 per patient to approximately £16,000 per MACI implant or £18,000 for a single vial of cells for ACI [18]. Therefore, in 2015 the technique was appraised by the National Institute for Health and Care Excellence (NICE) in the UK for the treatment of articular cartilage defects of the knee. The conclusions of this appraisal were that, while short-term clinical benefits were observed, the long-term clinical efficacy remained uncertain and the technique did not have robust evidence to demonstrate cost-effectiveness. Further research and evidence were recommended. The cost-effectiveness conclusions were considered harsh and were addressed in a consensus statement by UK knee surgeons, who drew attention to the estimated cost-effectiveness of ACI being between £7000 to £100,000 per Quality-Adjusted Life Year (QALY) (with the NICE threshold set at £20,000–30,000 per QALY).

There is a relative paucity of users, with, for example, only ten in the United Kingdom, which makes the development of large-scale research a challenge. MACI remains a potentially fruitful avenue for symptomatic therapy in early cartilage disease and traumatic cartilage lesions, though crucially not in OA.

## Medical approaches

The scope of ‘non-surgical’ alternative therapies is large and, for this reason, this review focuses on the treatments which are likely to arise in clinical discussion with OA patients including autologous MSC injection, PRP, vitamin D and ‘other’ treatments.

### Autologous mesenchymal stem cells

Articular cartilage is formed of a single cell type, the chondrocyte and a stable extracellular matrix that has no vascular, lymphatic or nervous supply. Subchondral bone provides mechanical and nutritional support and microfractures in this tissue can result in the release of undifferentiated mesenchymal stem cells (MSCs) from the bone marrow to facilitate the repair of chondral defects.

**Fig. 2:**
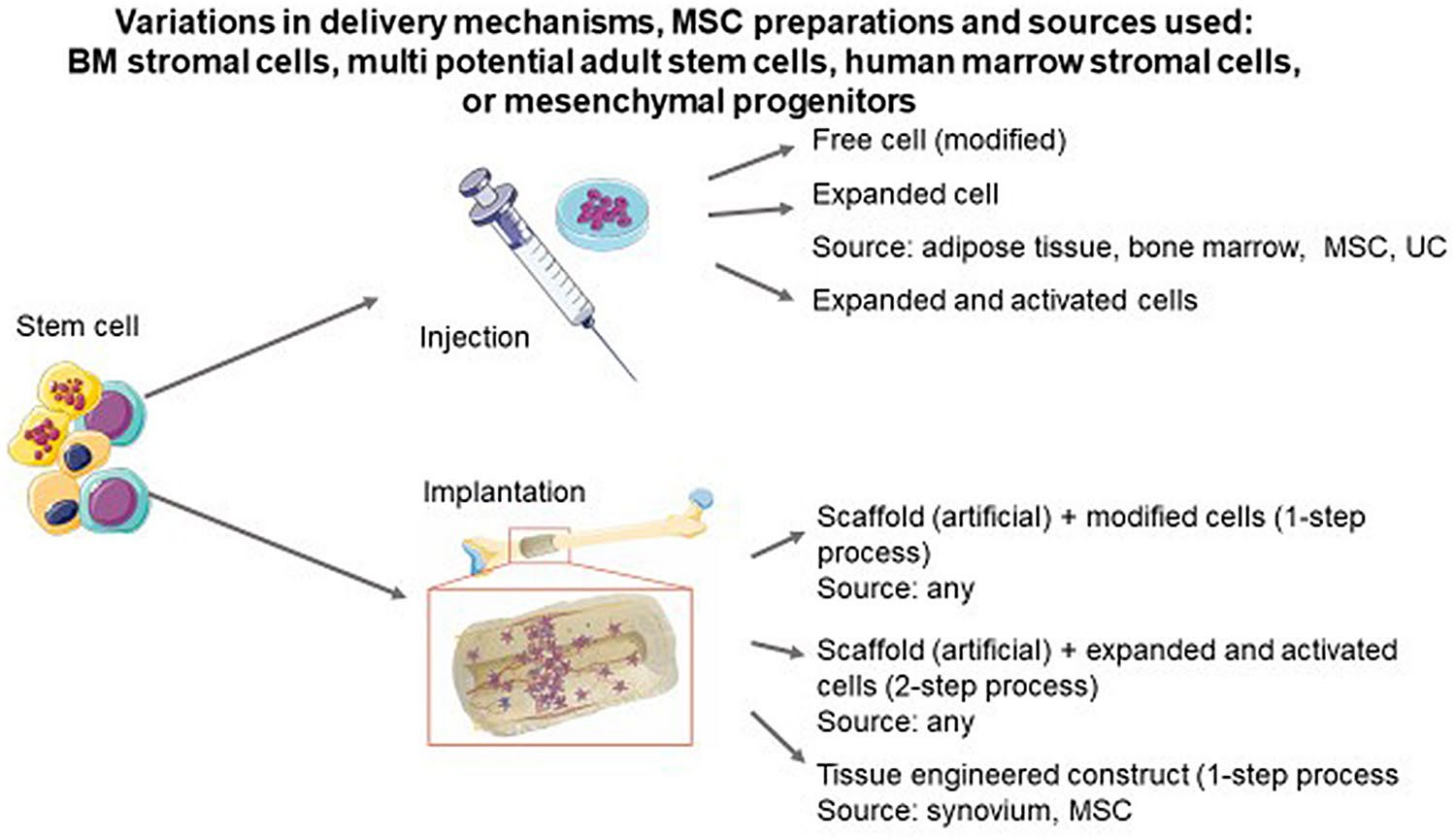
A depiction of the injectable and implantable options for delivery of MSCs and the potential sources of MSCs which are appropriate for each method

The repair capacity of MSCs has led to the development of techniques to directly inject MSCs locally into the joint following the ex vivo preparation of mesenchymal cells (Fig. 2). MSCs (also known as human or bone marrow stromal cells, multi-potent adult stem cells, mesenchymal progenitor cells and skeletal stem cells) have the ability to differentiate into the three tissue types, cartilage, bone and fat, and are invested, by definition, with an innate capacity for self-renewal and rapid proliferation. MSCs display paracrine anti-inflammatory and immunomodulatory properties [19, 20] and can be harvested from bone marrow (biopsy or aspirate) as well as from the stromal vascular fraction in adipose tissue. (Table 1) Autologous sources avoid any immunological concerns, however, **t**here is concern that, as MSCs are cleared from the joint rapidly, the joint may simply be experiencing the benefits afforded by a ‘wash-out’.

**Table 1:** A summary of the advantages and disadvantages in OA therapy of MSCs acquired from different sources, including; bone marrow, adipose tissue and stromal vascular fraction, the synovial membrane, umbilical cord and peripheral blood, synovial fluid and amniotic fluid

MSCs Cell source	Advantages	Disadvantages
Bone marrow (bone marrow conc. and bone marrow aspirate conc.)	High chondrogenic potential Relative ease of collection	High variability in MSC number MSC numbers and quality decline with age
Adipose tissue and stromal vascular fraction	Ease of harvest Large amount of tissue can be extracted Limited donor site morbidity	MSC numbers decline with obesity Lower chondrogenic potential
Synovial membrane	High chondrogenic potential Lowest osteogenic potential among MSCs	Limited number
Umbilical cord MSCs	Major source of allogeneic cells Ease of harvest Unlimited numbers	Chondrogenic potential variable
Peripheral blood, synovial fluid, amniotic fluid	Relative ease of collection	High variability in MSC numbers Chondrogenic potential variable

The use of MSCs in OA is an area of burgeoning research and, at the time of writing, there were 182 studies recorded, in various states of progress and 3 systematic reviews which best address the use of stem cells in the treatment of OA [21−23].

The review performed by Hached and colleagues analysed a total of 44 trials of intra-articular injection of MSCs in the treatment of OA including bone marrow-derived, adipose tissue-derived and umbilical cord MSCs [21]. The review concluded that all three methods of acquisition of MSCs had evidence to support their use in the treatment of OA and that intra-articular injection of these cells was safe with very few side effects. An extensive review appraised 20 full-text records including systematic reviews, comprehensive reviews, clinical reviews and meta-analyses published between 2006 and 2016 which addressed the treatment of cartilage lesions with MSCs [23]. In their review, the authors noted that improvements in symptoms (pain and function) were more commonly reported than structural/tissue improvement. There was a low level of evidence for the intervention with a mere 25 items of Level I graded evidence and subsequently concluded that it was “unclear” if stem cells were an effective treatment for OA. Broadly, stem cell therapies were effective in symptomatic (pain) relief related to chondral defects and defects (or lesions) due to OA. The authors reported, overall, limited repair and integration with extensive variability in the results presented. The main issues recorded included significant variability in MSC sources, techniques for preparation, methods of administration and the range of co-interventions used (including micro-fracture, sub-chondral drilling, debridement, PRP).

There are very few studies which have demonstrated any degree of structural improvement in knee OA. Lamo-Espinosa and colleagues [24], reported the results of an RCT of increasing doses (10 × 10^6^ or 100 × 10^6^) of bone marrow MSC, intra-articular injection against hyaluronic acid injection in 30 patients with OA (Kellgren–Lawrence grades II–IV). Participants were followed-up for 12 months and those in the MSC injection group had significant improvements in functionality and symptoms. Interestingly, only those in the high dose MSC injection group had statistically significant structural improvement in cartilage thickness on MRI at 12 months, opening the possibility of a dose–response. It should be noted that the inclusion of patients with such severe disease (Kellgren–Lawrence IV) suggests that the experimental group was substantially heterogeneous.

A pilot study by Orozco and colleagues [25] performed on patients with mild to severe knee OA (Kellgren–Lawrence grades II–IV) who received an intra-articular injection of (40 × 10^6^) bone marrow-derived MSCs, demonstrated improvement in pain, function and cartilage quality at 12 months. The pain relief was maintained at 2 years, while the objective cartilage improvement (on MRI) continued on a trajectory of improvement at 2 years [26]. However, it must be noted that this study only included 12 patients and so conclusions should be tentative at best.

Allogeneic MSC injection was demonstrated to be both feasible and safe, as reported in previous studies [27], though the observed negative outcomes include the generation of fibrocartilage, injection-related pain and swelling, infection post-bone marrow aspirate and a pulmonary embolus 2 weeks post-bone marrow aspirate [23]. In a further systematic review, only 2 serious adverse events (synovial effusion and unstable angina) were observed amidst 288 patients.

Pers and colleagues studied three dosages of adipose tissue-derived MSCs (2 × 10^6^, 10 × 10^6^, 50 × 10^6^ cells) in the Adipose Derived mesenchymal stromal cells in Patients with knee Osteoarthritis (ADIPOA) trial and found 2 × 10^6^ was optimal in terms of functionality and pain relief at 9 months [28] and postulated MSCs may operate via innate and adaptive immune modulation [29]. This phase I trial is now in phase II (ADIPOA-2) and recruitment of 150 patients is underway.

Limitations and barriers to the routine application of MSCs for OA from this plethora of clinical studies were a consequence of (i) significant variation in MSC source, (ii) significant variation in MSC preparation protocols adopted, (iii) significant variation in MSC delivery approaches adopted and, (iv) significant variation in the number of different co-interventions with MSCs including micro-fracture, sub-chondral drilling, debridement, and PRP as well as hyaluronic acid, albumin and serum, osteophyte removal, and surgical interventions (ACL repair and high tibial osteotomy).

Nevertheless, the following factors were associated with increased efficacy of MSC injection for OA:

- Younger age
- Male gender
- Low BMI
- Small lesion/defect
- Early/mild to moderate OA severity

It should also be considered that these MSC treatments are currently not covered by many health insurance providers and the costs are high. For example, in the United States, the cost of a single stem-cell treatment for osteoarthritis was estimated at $5156 (95% CI $4550–5762) based on data from 273 centers [30].

In conclusion, the predominantly poor-quality, current literature suggests that symptoms, particularly pain, may improve with MSCs injection, however, evidence of structural improvement is unconvincing and positive effects appear to be observed in particular patient phenotypes. The overwhelming conclusion is a need to standardize the intervention if progress is to be made (Table 2). There is also a substantial need for phase II and III trials with the results of ADIPOA-2 being keenly awaited.

**Table 2:** A synthesis of the main issues in MSC efficacy in the treatment of OA and limitations to adequate assessment through clinical trials

Key factors for stem cell efficacy in OA	Key factors limiting stem cell efficacy and clinical trial interpretation of MSC use in OA
• Age (younger patients typically display better outcomes) • Gender (males typically better outcomes compared to female counterparts) • BMI (lower BMI is associated with better outcomes) • Lesion or defect size (better repair associated with smaller lesion size) • Stage of OA (early OA, mild to moderate OA correlated with better outcomes)	• Significant variation in MSC source • Significant variation in MSC preparation protocols • Variation in MSC Delivery • Significant variation in the number of different co-interventions with MSCs Micro-fracture, sub-chondral drilling, debridement, and platelet rich plasma Less common—hyaluronic acid, albumin and serum, osteophyte removal, and surgical interventions (ACL repair and high tibial osteotomy)

### Platelet-rich plasma

Platelets play an important role in coagulation but also inflammation and PRP is a therapy which has been used extensively in equine tendinopathy [31] and has been investigated in the treatment of OA, particularly of the knee [32].

Platelet-rich plasma is a fluid which is rich in growth factors that stimulate cell proliferation, cellular migration, angiogenesis and the synthesis of the extracellular matrix including; platelet-derived growth factor (PDGF), tumor-like growth factor-β (TGF-β), fibroblast growth factor (FGF), vascular endothelial growth factor (VEGF), hepatocyte growth factor (HGF) and insulin-like growth factor-1 (IGF-1).

**Fig. 3:**
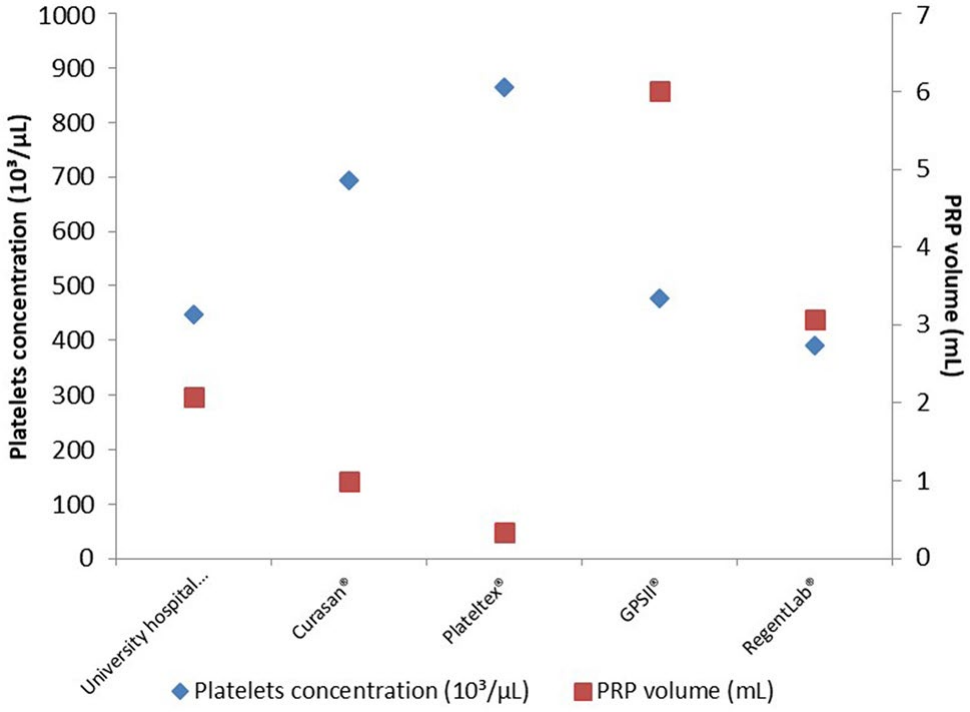
A comparison of platelet concentration and volume of protein-rich plasma (PRP) resulting from the extraction methods employed by 5 laboratories. [33]

It is derived through centrifugation of a patient’s blood, with the aim of separating a plasma component which is rich in platelets (> 95% platelets) from whole blood which is poor in platelets (4% platelets). The PRP is then extracted and injected into the affected joint. The intricacies of preparation techniques vary and result in significantly different constituent cells (erythrocyte and leucocyte proportions), platelet concentrations and injection volumes [33] (Fig. 3). Indeed, there is a global schism in practice with Europeans preferring to use leukocyte-poor and Americans using leukocyte-rich PRP. PRP has been investigated in RCTs [34, 35] but the broad variation in preparation methods makes inter-trial comparison difficult and robust conclusions harder to ascertain and few are blinded. To emphasize this point we have synthesized and summarized some of the seminal studies below.

The issues surrounding the preparation of PRP are covered in a review of the techniques utilised in a number of RCTs and systematic reviews [36]. There is substantial variation in techniques including; the subject studied (severity of knee OA), PRP preparations, the inclusion of leukocytes, platelet count, number of injections delivered, interval/frequency of administration, volume of injection, whether fresh or freeze-thawed PRP were used, the use of anticoagulants and activating agents, separation techniques and any co-administered injections. With this in mind, a technical analysis was performed in 2017 to evaluate the similarities and differences between differing PRP formulations, in an attempt to determine the best preparation for the treatment of knee OA.

Filardo and colleagues [37, 38] performed a blinded trial in which they recruited participants with radiographic knee OA up to a Kellgren and Lawrence score of ≤ III, with 96 randomised to PRP and 96 to hyaluronic acid as a comparator. The PRP was centrifuged twice and PRP participants received 3 injections, once a week for three weeks and all participants were followed up for 12 months initially but extending to 5 years [39]. The key finding was that both treatments were equally effective in reducing knee OA symptoms and improving function over time but leucocyte-rich PRP was no more effective than hyaluronic acid.

To summarize the available evidence regarding PRP a number of systematic reviews have been performed [40−42]. PRP provided significant improvements in knee OA patient outcomes at 12 months and larger improvements were observed in those with milder radiographic disease (Kellgren and Lawrence ≤ II) [40],

Significant improvements in ‘patient recorded outcomes’ were also observed with PRP as opposed to hyaluronic acid at 3–6 months (WOMAC 28.5 vs. 43.4 respectively, *p* = 0.0008) and 6–12 months (WOMAC 22.8 vs. 38.1, *p* = 0.0062) [41].

A further systematic review published in 2018 (including 7 randomized placebo-controlled trials and 908 patients) sought to investigate the superiority of PRP over hyaluronic acid which was not demonstrated. In respect of PRP the minimal clinically important difference (MCID) was observed in 5 of the 7 papers, and suggested that differences in clinical outcomes could be due to variation in the preparation of PRP in terms of; centrifugation (speed, frequency, time-length, activating agents), administration (frequency, volume of injection) and post-administration rehabilitation protocols [42]. From a safety point of view, no local or systemic serious adverse events were noted in the reviewed articles.

Milants and colleagues used a previous definition of minimal clinically important improvement in pain (MCII) to determine whether an observed difference had any ‘meaningful’ effect in clinical practice. This was set at 15 out of 100 for absolute improvement and 20% for relative improvement for knee OA, as defined by Tubach et al. [43].

The Milants technical analysis included 19 RCTs, and studies were classified into two groups depending on outcomes with a ‘bad responder group’, defined as a response less than the minimal clinically important improvement (MCII) (*n* = 4 studies), and a ‘very good responder group’, defined as a response greater than twice the MCII (*n* = 7 studies). The reviewers contacted authors of the trials to obtain information regarding the preparation which was missing from the manuscript and PRP preparation was classified according to the Mishra (a classification in which PRP is divided into 4 types depending on 3 variables; white blood cells: increased or minimal, activation: yes or no, platelet content > 5 times patient baseline or ≤ 5 times patient baseline) and PAW (Platelet concentration, Activation prior to injection, White blood cell content).

In almost all studies with a very good responder group, PRPs were leukocyte-poor, activated prior to injection and platelets < 5 times baseline or between baseline and 750,000 platelets/µL), administered according to a lower number of injections (1 or 2 rather than 3), with a longer interval between injections (2 to 3 weeks per injection rather than once weekly) and a single (as opposed to double) spinning technique. The use of leukocyte-rich PRP was only found in the bad responder group. The use of calcium chloride and citrate was common in the very good responder group.

The cost of the PRP procedure is estimated at $714 (95% CI $691–737) based on data from 179 centers from across the United States [44].

In conclusion, although PRP may have repeated mild symptomatic benefits, there is yet to be experimentally robust demonstration of symptomatic and structural effects in the current literature. Research is required to better understand the mechanism of action, including investigation of the survival and location of platelet-derived factors within the joint following injection. In order for PRP to be considered within the dogma of recommended treatment for OA, at least one large, randomized, placebo-controlled trial and further investigation regarding preparation and dosage efficacy is required. This working group cannot, therefore, make a recommendation to use PRP as an intervention for OA.

### Vitamin D

There is a secular trend toward decreased vitamin D levels, with serum concentrations averaging 49 ng/mL in the mid-twentieth century to approximately 23 ng/mL now, and with over a billion individuals being vitamin D deficient or insufficient [45]. Due to the role played by sunlight in the in vivo production of vitamin D, particularly low levels are observed at the extremes of latitude [46], and in winter months. Studies of seasonal gene expression have shown that some pro-inflammatory factors, including soluble IL-6 receptor and C-reactive protein have a peak expression in winter months and vitamin D receptor expression peaks in the summer months [47]. It is, therefore, interesting that certain diseases display similar seasonality and geography, including OA. This descriptive epidemiological observation is supported by basic scientific findings including [48]:

- There are receptors for vitamin D on chondrocytes which may play a role in the regulation of matrix metalloproteinases and prostaglandin E2 production
- Vitamin D stimulates proteoglycan synthesis in mature chondrocytes
- Vitamin D deficiency influences bone remodeling which may predispose to the development of OA

Despite these observations, four RCTs of vitamin D in OA have been performed in the United States (US) [49], India [50], the United Kingdom (UK) [51] and Australia [52]. None of these have demonstrated structural or symptomatic benefit in OA.

The pilot study performed in India [50] included 103 participants (59.4% females) with a baseline age of approximately 50 and a baseline 25-OH vitamin D of < 20 ng/mL. They found a significant reduction in knee pain and improvement in function but no significant alteration in radiographic knee OA at 12 months.

The placebo-controlled trial performed by McAlindon and colleagues [49] (in the US) included 146 female participants with a mean age at baseline of 62.4 years. At two years they found no improvements in knee symptoms, functional status or cartilage structure with vitamin D.

In the aforementioned UK placebo-controlled trial [51], despite an increase in serum 25-hydroxy-vitamin D (from approximately 20 to 30 µg/L) in the treatment group, no significant changes in symptomatic or radiographic knee OA were observed after 3 years in 474 participants (over the age of 50).

In Australia, Jin and colleagues demonstrated that in 209 patients with low vitamin D (12.5–60 nmol/L) treated for 2 years with monthly oral vitamin D3 (50,000 IU), there was no significant improvement in MRI-measured tibial cartilage volume or WOMAC knee pain score [52].

The relationship between vitamin D and knee OA has been investigated in a recent systematic review of 11 studies, which concluded that although vitamin D deficiency is associated with knee OA, the evidence regarding this association is inconsistent [53]. The studies included in the review were largely of cohort and cross-sectional design but also included two RCTs. The systematic review demonstrated that there was marked variation in the relationships between vitamin D and OA with a level of evidence (for an association of vitamin D deficiency with prevalent symptomatic knee OA) of ‘moderate’, while the relationship with prevalent radiographic knee OA was graded as ‘limited’. This negative conclusion supports that of a prior systematic review [54].

It should be acknowledged that vitamin D deficiency has been associated with a range of co-morbidities, of which OA is only one. However, the adverse effect profile of the supplement is favorable and should be strongly considered in those at risk of deficiency. Should there be systematic screening for vitamin D deficiency or systematic supplementation of vitamin D? This question is beyond the purview of this article and is dependent on many factors which are related to local healthcare systems and economic considerations.

We conclude by recommending that, when severe deficiency is diagnosed (especially in winter), vitamin D should be supplemented through the evidence that such supplementation ameliorates OA symptoms is inconclusive.

### Other medical therapies

It should be noted that the medical therapies included in this section are very rarely mentioned in international guidelines, however, they are often the subject of discussions between patients and clinicians. Collagens, methylsulfonylmethane, *S*-adenosylmethionine, curcuma, harpagophytum and ginger are commonly used in the treatment of OA in many countries [55], with polyphenols, green tea, ‘Cat’s claw’ and dairy products also being mentioned.

#### Collagens: oral and intra-articular

Oral collagens are a rich source of amino acids, and, in OA, are purported to stimulate the joint to produce endogenous collagens in response to supplementation.

In 2016 a study investigated 190 patients, randomised to receive undenatured type II collagen (40 mg) or glucosamine hydrochloride and chondroitin sulfate or placebo [56]. The primary outcome was total WOMAC change from baseline with secondary outcomes being Lequesne index and pain VAS. After 6 months they found that undenatured type II collagen led to a significantly greater reduction in WOMAC compared to placebo (551 vs. 414, *p* = 0.002) and compared to the glucosamine and chondroitin sulfate arm (551 vs. 454, *p* = 0.009). In terms of secondary outcomes, there was a greater reduction in Lequesne index (2.9 vs. 2.1, *p* = 0.009).

A further RCT investigated the performance of 5, once weekly 4 mL injections of polymerized collagen type I (of porcine origin) compared to sodium hyaluronate with assessments at 3 and 6 months [57]. The primary outcome was Lequesne index (measuring the severity of knee OA) at the 3 month time point with a visual analogue score for pain and SF-36 questionnaire also recorded. They found no significant differences between the groups for the above outcomes at either 3 or 6 months.

Gelatin, a form of collagen-rich in proline, was assessed in 52 patients as part of a randomised, placebo-controlled trial which found significant inter-group differences in several types of pain, however, an effect size was not reported, making extrapolation to clinical benefit difficult [58]. Undenatured collagen, an alternative form of collagen, was compared to glucosamine and chondroitin with 26 patients per group. This found no inter-group differences in the efficacy of the interventions [59].

In 2012, Van Vijven and colleagues published a systematic review of a variety of oral collagens at various doses comprised of 8 trials of collagen hydrosylates (3 versus placebo), gelatin (1 versus placebo) or undenatured collagen (versus glucosamine hydrochloride and chondroitin sulfate) [58]. The review concluded that there was ‘low’ grade evidence for the use of these in OA. Those treated with collagen hydrosylates included 313 treated patients (taking 10 g per day) against 297 on a placebo preparation and found a significant effect on symptoms (WOMAC pain (− 0.48) with a significant but small effect size of 0.17) but no effect on the joint structure as assessed by MRI scan.

There are various preparations of oral or intra-articular collagens. Although widely used in a large number of countries, current data do not support a positive recommendation to treat OA patients despite a mild effect on symptoms (pain) and function.

#### Methylsulfonylmethane (MSM)

This dietary supplement is found in plants, fruits and vegetables, and can be taken alone or in combination with other supplements. There have been two, notable, placebo-controlled trials of MSM in patients with knee OA, both in approximately 50 patients over 12 weeks of follow-up. The first [60] involved a dosage schedule which resulted in 6 g per day and demonstrated a significant improvement in SF-36, WOMAC pain and function in the MSM group (*p* < 0.05). The second involved a dose of approximately 3 g per day demonstrated a significant improvement in WOMAC function but not in WOMAC pain or SF-36 compared to placebo [61].

A trial in knee OA randomised 118 patients between glucosamine, MSM, combination therapy or placebo and found a significant improvement in pain and Lequesne functional index at 12 weeks in all groups except the placebo arm.

A recent RCT examined the performance of glucosamine and chondroitin in combination with MSM versus glucosamine and chondroitin alone and versus placebo, in a population of 147 early knee OA patients (Kellgren and Lawrence grade I-II). This study demonstrated improvement in the groups which included MSM compared to the other treatment groups, in terms of pain VAS and WOMAC scores [62].

In conclusion, small trials did not demonstrate any major safety concerns for MSM treatment. Whether there is a symptomatic benefit over a short follow-up period is a question which would need to be answered through larger, well-designed trials and long term follow-up data are required.

#### S-adenosylmethionine (SAMe)

This is a substance produced from methionine in the liver. The treatment of hip and knee OA with SAMe was the subject of a Cochrane review of 4 randomised, placebo-controlled trials [63]. This review included 656 patients and demonstrated a significant improvement with SAMe compared to placebo but with a very minor effect on pain (4 mm on a 100 mm VAS) and function (2 mm on a 100 mm VAS), which are of questionable clinical significance. There was no significant difference in adverse effects or withdrawals but it should also be noted that the methodological and reporting quality were poor and that there was a moderate degree of inter-trial heterogeneity (*I*^2^ = 54%).

#### Curcuma

Curcuma (or curcumin) is an extract of turmeric, a yellow spice, and member of the *ziangiberaceae* family. Both curcuma and ginger have roots in Ayurvedic and Chinese medicine [64] with curcuma manifesting an anti-inflammatory effect via cyclo-oxgenase (COX)-2, prostaglandins and leukotoxin inhibition. There is a wide variation in daily doses from 180 to 2000 mg, which makes direct, inter-study comparison problematic. Nevertheless, a meta-analysis was published in 2016 included 4 placebo-controlled trials in the context of knee OA; 2 trials versus ibuprofen and 1 versus diclofenac. In these trials of curcuma, improvements were seen in symptomatic measures and NSAID consumption (up to 4 months) versus placebo but no significant superiority was noted versus ibuprofen or when added to diclofenac.

Although a few, idiosyncratic adverse effects were reported in the trials, the meta-analysis concluded that curcuma could be considered safe at daily doses of 4800 mg for 4 months [65].

A more recent meta-analysis, published in 2018, included 11 RCTs (*N* = 1009 patients) investigating the role of curminoids and boswellia (a gum-resin used in Ayurvedic medicine) in the treatment of knee OA [66]. There was some improvement in pain and function outcomes versus placebo, however, the conclusion was that evidence was currently too scant to allow the therapies to be included in clinical recommendations for treatment.

A trial of bio-optimized curcuma in the treatment of knee OA was reported in 2019 [67]. After 3 months of treatment, there were no statistical differences in intention to treat analyses for curcuma efficacy comparing the treatment to placebo arms for the co-primary endpoints (which are not included in those recommended by the European Medicines Agency or scientific societies). However, there was a significantly higher incidence of diarrhea in the curcuma group. In summary, experimental data regarding curcuma is sparse. The little evidence there supports a mild effect on OA symptoms. The sample sizes of the published trials are small, follow-up is short [65] and longer-term, robust studies are required before curcumin can be actively recommended from an efficacy stand-point.

#### Harpagophytum

Also known as ‘Devil’s claw’ Harpagophytum is an African plant which is thought to manifest anti-inflammatory effects, similar to curcuma, via inhibition of COX-2 and leukotoxin. A systematic review by Gagnier and colleagues investigated the role of Harpagophytum in the treatment of lower back pain and OA, including 3 randomised, placebo-controlled trials of hip and knee OA (385 patients) [68]. This review concluded that there was ‘moderate’ evidence of effectiveness for the use of 60 mg of harpagophytum powder, though longer, higher-quality trials are required before it can be routinely recommended in clinical practice.

#### Ginger

Ginger, another member of the *ziangiberaceae* family, is thought to manifest anti-inflammatory effect via inhibition of COX, lipoxygenase, reduction in tumor necrosis factor and inflammatory prostaglandin production. There are data to suggest that ginger extract (Zintona EC®) is superior to placebo in terms of pain relief at 6 months (though efficacy was the same at 3 months) in a double-blind, randomised, placebo-controlled, crossover trial of knee OA [69].

Additionally, trials have demonstrated improvements in pain and mobility and reduced rescue medication usage intake versus placebo, though with more (mostly mild) gastrointestinal adverse events [70].

In 2015, the trial data were summarized and analyzed in a systematic review and meta-analysis of 5 randomised placebo-controlled trials, totaling 593 patients, which found a significant reduction in pain and disability with ginger. However, twice the rate of discontinuation was observed with ginger versus placebo (Relative Risk 2.33, 95% CI 1.04–5.22) [71]. A similar finding was reported in an earlier systematic review which noted “infrequent reports of mild, and predominantly gastrointestinal, adverse effects” [72].

## Conclusion

In this review, we have synthesized the current evidence regarding alternative therapies for OA. Our findings are summarised in Table 3. Publication bias may be an issue with this group of treatments, however, this is not always the case (as seen throughout this review).

**Table 3:** A summary table of the key, take-home points for each of the interventions reviewed

Alternative therapy	Key points
Autologous chondrocyte implantation	• Treatment for cartilage defects and not osteoarthritis • Includes ACI and MACI • Trial evidence to support symptomatic benefit • Supported by NICE in the UK for specific patient group (including no previous knee surgery, limited evidence of knee osteoarthritis, large chondral defect)
Mesenchymal stem cell injection	• There are multiple sources of MSCs with differing profiles of usage and limitations • Multiple sources of MSCs lead to difficulties in directly comparing clinical trials • Trial evidence to support symptomatic benefit • Limited evidence to support structural benefit (MRI cartilage thickness)
Platelet-rich plasma	• Trial evidence to support symptomatic benefit • Multiple methods of preparation lead to difficulties in directly comparing clinical trials
Vitamin D	• Evidence of efficacy in clinical trials is equivocal • Recommendation to provide supplementation to those patients with evidence of depleted levels of 25OH-vitamin D
Other alternative therapies	• Very limited clinical trial data to suggest the efficacy of oral collagens, MSM, SAMe, curcuma and ginger • Adverse events: largely rare, though ginger appears to be associated with an increased risk of mild gastro-intestinal adverse events

In summary, for all of the interventions covered in this review, issues of study design limit the degree to which inference can be made about clinical effectiveness in symptomatic OA. It is clear that none of these would currently clear the required hurdle for regulatory approval, were they to be assessed in like manner to current pharmaceutical interventions. However, there is also an insufficient basis for declaring them completely ineffective. They, therefore, remain an area in which further, appropriately designed, large, blinded, RCTs are an urgent necessity.

## Electronic supplementary material

Below is the link to the electronic supplementary material.

Supplementary file1 (DOCX 59 kb)
