# Supplementary material for: Alternative and complementary therapies in osteoarthritis and cartilage repair
Source: Aging Clin Exp Res. 2020 Mar 13;32(4):547–60. doi: 10.1007/s40520-020-01515-1 (PMC7170824; doi:10.1007/s40520-020-01515-1)
Supplement: Supplementary file 1 — Supplementary file1 (DOCX 59 kb) [file 40520_2020_1515_MOESM1_ESM.docx]

**Supplementary table 1** A summary of the randomised controlled trials, systematic reviews and meta-analyses included for each alternative treatment

| Treatment | Randomised controlled trials | Reviews | Meta-analyses |
| --- | --- | --- | --- |
| Autologous chondrocyte implantation (ACI) | [^8^](#_ENREF_8),[^9^](#_ENREF_9) | [^10^](#_ENREF_10) |  |
| Matrix ACI | [^14^](#_ENREF_14), [^15^](#_ENREF_15) |  |  |
| Mesenchymal stem cells | [^24^](#_ENREF_24), [^25^](#_ENREF_25), [^28^](#_ENREF_28), [^27^](#_ENREF_27) | [^21-23^](#_ENREF_21) |  |
| Platelet-rich plasma | [^34^](#_ENREF_34), [^35^](#_ENREF_35), [^37^](#_ENREF_37),[^38^](#_ENREF_38), [^39^](#_ENREF_39) | [^73^](#_ENREF_73), [^40^](#_ENREF_40), [^41^](#_ENREF_41), [^42^](#_ENREF_42) |  |
| Vitamin D | [^50^](#_ENREF_50), [^49^](#_ENREF_49), [^51^](#_ENREF_51), [^52^](#_ENREF_52) | [^53^](#_ENREF_53)^,^ [^54^](#_ENREF_54) |  |
| Collagens | [^56^](#_ENREF_56), [^57^](#_ENREF_57), [^59^](#_ENREF_59) | [^58^](#_ENREF_58) |  |
| Methylsulfonylmethane | [^60^](#_ENREF_60), [^61^](#_ENREF_61), [^62^](#_ENREF_62) |  |  |
| S-adenosylmethionine |  | [^63^](#_ENREF_63) |  |
| Curcuma |  | [^65^](#_ENREF_65) | [^65^](#_ENREF_65), [^66^](#_ENREF_66) |
| Harpagophytum |  | [^68^](#_ENREF_68) |  |
| Ginger | [^69^](#_ENREF_69) | [^71^](#_ENREF_71) | [^71^](#_ENREF_71) |
